# Regulatory role of epigenetics in rice immunity against bacterial and fungal pathogens

**DOI:** 10.1007/s44297-026-00080-9

**Published:** 2026-07-01

**Authors:** Gulmeena Shah, Habib Ullah, Lin Chen, Amir Zaman Shah, Rozina Shaheen, Jinglan Liu, Weiwen Kong

**Affiliations:** 1 College of Plant Protection, Yangzhou University, Yangzhou, Jiangsu China; 2 Joint International Research Laboratory of Agricultural & Agri-Product Safety, Jiangsu Key Laboratory of Crop Genomics and Molecular Breeding, The Ministry of Education of China, Yangzhou University, Yangzhou, Jiangsu China

**Keywords:** Epigenetic regulation, Rice immunity, Disease resistance, DNA methylation, Histone modification, Non-coding RNAs

## Abstract

Rice (*Oryza sativa*), a major crop, faces significant threats from various pathogens, which affect global food security. Recent research in plant biology highlights the crucial role of epigenetic mechanisms in regulating rice immunity. Epigenetics involves heritable changes in gene expression without alterations to the DNA sequence, mediated by DNA methylation, histone modifications, and non-coding RNAs. This review critically evaluates the role of epigenetics in rice immunity, focusing on key epigenetic modifications and their impact on disease resistance, including *WRKY* transcription factors and epigenetic regulatory factors, such as RdDM (RNA-directed DNA methylation). These epigenetic regulators enable rice plants to dynamically withstand pathogen attacks by modulating the expression of defense-related genes. This review evaluates the epigenetic responses of rice to major pathogens, including *Magnaporthe oryzae* (*M*. *oryzae*) and *Xanthomonas oryzae* (*Xoo*). We move beyond simple descriptions of epigenetic marks to analyze the functional integration of these pathways and their trade-offs with plant growth. Finally, we discuss the potential of "epigenetic breeding" and CRISPR-based epi-editing as a sustainable frontier for developing broad-spectrum resistance in rice. Future research should focus on identifying specific epigenetic markers associated with resistance traits and integrating epigenetic approaches with traditional breeding and biotechnological methods to achieve sustainable rice production and ensure food security in the face of evolving pathogen threats.

## Introduction

### Broader implications and applications

Epigenetics encompasses heritable changes in gene function independent of DNA sequence alterations. In plants, epigenetic regulation plays an essential role in controlling gene activity in response to environmental stimuli, including pathogen attack. The major epigenetic mechanisms include DNA methylation/demethylation, histone modification, chromatin remodeling, and small RNA-mediated regulation, all of which influence chromatin structure and transcriptional activity in response to pathogen attacks [1].

DNA methylation typically silences genes unnecessary for immediate defense and conserves energy [2]. Histone modifications such as methylation and acetylation alter chromatin structure, regulating accessibility for transcriptional machinery [3]. While non-coding RNAs target specific microRNAs (miRNAs), and small interfering RNAs (siRNA) modulate gene expression at the transcriptional and post-transcriptional levels [4].

These mechanisms collectively enable rice plants to rapidly or reversibly respond to pathogen challenges by fine-tuning defense-related gene expression that affects host–pathogen interactions [5, 6]. Such dynamic regulation allows rice plants to balance growth and immunity under fluctuating environmental conditions [7, 8].

The current review of epigenetic regulation in rice immunity provides a foundation for understanding plant-pathogen interactions [9, 10]. Rapidly occurring and heritable epigenetic changes allow rice to adapt quickly to new threats and offer new opportunities for crop improvement [11–15]. Detailed mechanisms and their specific role in disease resistance against *Xoo* and *M*. *oryzae* are discussed in the following sections.

## Key epigenetic mechanisms regulating rice immunity

### Epigenetic regulation of pathogen resistance

In rice, epigenetic regulation contributes to disease resistance through dynamic changes in DNA methylation and chromatin states in response to pathogen pressure [16]. These modifications regulate the expression of defense-related genes and help maintain a balance between growth and immune responses [17]. In rice, DNA methylation occurs in CG, CHG, and CHH sequence contexts and is regulated by key enzymes, including Chromomethylase3 (CMT3), which mediates CHG methylation (H is A, C, or T), Domains Rearranged Methyltransferases (DRM), which catalyze CHH methylation; and DNA Methyltransferase 1 (MET1), which catalyzes CG methylation, a common sequence context in plants [18, 19]. These methylation patterns are maintained and modulated through Semi-conservative mechanisms that preserve CG and CHG methylation patterns and RdDM, which is guided by siRNAs [20, 21].

**Fig. 1:**
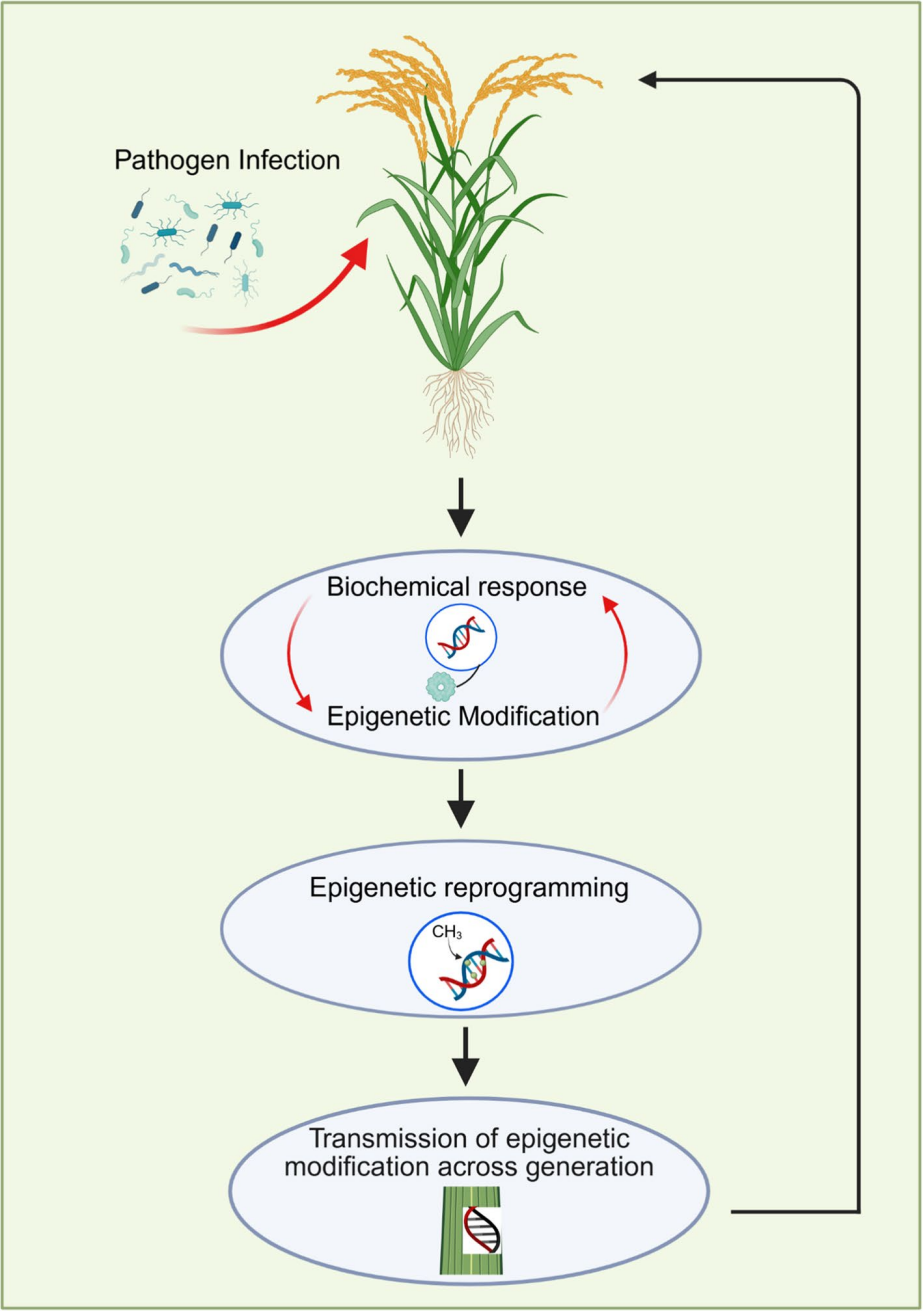
Epigenetic reprogramming of transgenerational stress adaptation in rice (Created in https://www.biorender.com)

RdDM plays an important role in regulating genome stability and plant immunity [22] by catalyzing de novo cytosine methylation, particularly at small transposable elements (TEs) or large TE borders, and it involves specific RNA polymerases, Pol IV and Pol V, independently producing short single-stranded RNAs at RdDM target loci [23]. Furthermore, RNA-DEPENDENT RNA POLYMERASE 2 (RDR2) generates double-stranded RNAs (dsRNA) from Pol IV transcripts, and DICER-LIKE 3 (DCL3) processes them into 24-nt siRNAs. ARGONAUTE 4 (AGO4) binds siRNAs and targets RdDM loci using Pol V transcripts as scaffolds. DRM2 is recruited via AGO4 interaction and catalyzes cytosine methylation [18]. Pathogen infection triggers rice plants to produce small RNAs that silence TEs, often linked to genomic instability and disease susceptibility [24]. Silencing these elements enhances resistance to pathogens, a trait potentially inherited by offspring in rice (Fig. 1) [25]. Pol IV, essential for 24-nt siRNA production in the RdDM pathway, has its largest subunit encoded by *OsNRPD1a* and *OsNRPD1b* in rice [26]. Knockdown of *OsNRPD1a/b* resulted in dwarfism, increased tillering, reduced panicle length [27], and disease resistance through an unknown pathway. The expression of two miRNA genes, *OsmiR156d* and *OsmiR156j*, which were differentially expressed in *osnrpd1a/b* mutants, increased and reduced CHH methylation at miniature inverted-repeat TEs (MITEs) in their promoters [28]. WRKY45 is a key transcription factor regulated by multiple epigenetic mechanisms, including DNA methylation, histone modifications, and small RNA-associated pathways, which collectively fine-tune its expression during immune responses and contribute to disease resistance regulation against bacterial and fungal pathogens. Through this multilayered regulation, WRKY45-1 and WRKY45-2 serve as important molecular hubs linking epigenetic control and transcriptional reprogramming in rice immunity against *Xoo*. These findings highlight the importance of RdDM in maintaining proper gene regulation during development and stress responses.

Histone modifications also contribute to regulating rice immunity. Histone methylation, for instance, can promote heterochromatin formation, a transcriptionally inactive DNA state [29], preventing the expression of susceptibility genes [30, 31]. Activating marks such as Trimethylation of H3 lysine 4 (H3K4me3) are associated with transcriptional activation, whereas repressive marks such as H3K27me3 are linked to gene silencing [32]. These chromatin states control the accessibility of defense-related genes and contribute to the stable regulation of immune responses under pathogen stress [33].

Non-coding RNAs, including miRNAs and siRNAs, are crucial for epigenetic regulation of rice disease resistance [34]. These small RNAs guide the modification of histones and DNAs, silencing non-essential defense genes. Specific miRNAs target the mRNAs of susceptibility genes, enhancing disease resistance [35, 36]. miR156 targets SBP/SPL transcription factors, influencing plant development and disease resistance [37]. Reducing miR156 activity in rice increases resistance to *Xoo* but decreases yield [38]. miR168 binds the 3´ UTR of AGO1 mRNA, cleaving it and reducing AGO1 protein levels [39]. The conserved miR168-AGO1 regulates antiviral and antifungal RNA interference [40]. Genetic research has shown that AGO18 has broad-spectrum antiviral activity [41]. In rice, AGO18 sequesters miR168, releasing AGO1 for antiviral action, while overexpression of AGO1a imparts resistance to *Xoo* [40]. Overall, multiple miRNA families contribute to rice growth [42], hormone metabolism [43], and biotic and abiotic defense responses [44].

Collectively, these epigenetic mechanisms enable rice plants to integrate environmental signals into the precise regulation of gene expression, thereby modulating disease resistance in response to pathogen challenges.

### DNA methylation/demethylation and disease resistance

In rice, pathogen infection alters DNA methylation patterns, leading to activation or repression of immune-related genes [45]. In addition to cytosine methylation, emerging evidence suggests that DNA adenine methylation (N6-methyladenine, 6 mA) may also occur in the rice genome, indicating an additional layer of epigenetic regulation; however, its role in rice immunity remains largely unexplored. For example, the DNA de novo methyltransferase gene OsDRM2 regulates defense responses against *M. oryzae* [19]. Similarly, REPRESSOR OF SILENCING 1a (*OsRos1a*) also plays a vital role in rice resistance against *Xanthomonas oryzae* pv. *oryzicola* (*Xoc*) causes bacterial leaf streak (BLS) by regulating gene expression and DNA methylation [46] (Table 1). Manipulating DNA methylation patterns can potentially enhance defense gene expression and improve disease resistance [47, 48]. The rice resistance gene *Xa21* confers defense against *Xoo* strains [49]. Xa21G, a related protein, was isolated from methylation-sensitive amplification polymorphism screening in a mutant line derived from germinating seeds treated with the DNA methylation inhibitor 5-aza-2’-deoxycytidine [50]. The Xa21G promoter was methylated in wild-type plants, resulting in undetectable transcripts, but accumulated in a mutant line with reduced cytosine methylation, indicating wild-type silencing.

**Table 1:** Rice disease resistance mediated by epigenetic regulators

Epigenetic Regulators	Locus ID	Epigenetic Mechanism	Pathogen/Disease	Functional role in disease resistance	Reference
*OsRDR2, OsDCL3a,* *OsAGO4a*	LOC_Os04g39160 LOC_Os01g68120 LOC_Os01g16870	SiRNA-guided DNA methylation by the RdDM pathway	*M. oryzae*/Rice blasts	Modulate the *Pigms* expression using MITE-derived siRNAs, which are essential for disease resistance	Deng et al. 2017 [96]
*OsTE-siR815*	LOC_Os08g10150	SiRNA-guided DNA methylation by the RdDM pathway	*Xoo/*Bacterial blight	Suppress WRKY45 defense-related gene expression and negatively regulate disease resistance	Zhang et al. 2016 [54]
*OsROS1a*	LOC_Os09g12660	Demethylation	*Xoc/*BLS	Modulate DNA methylation patterns and influence immune-related pathways to enhancing disease resistance	Xie et al. 2025 [46]
*JMJ704,* *JMJ705*	LOC_Os05g23670 LOC_Os01g67970	Histone demethylation	*Xoo/*Bacterial blight	Positively regulate rice immunity by repressing negative regulators (*NRR*, *Os-11N3* and *OsWRKY62*) of plant defense	Hou et al. 2015 [62] and Li et al. 2013 [63]
*HDT701* *HDA705*	LOC_Os05g51830 LOC_Os08g25570	Histone deacetylation	*Xoo/*Bacterial blight *Uv/*False smut	Repress defense gene expression by regulating H4 acetylation of the PRR Remove lysine 2-hydroxyisobutyrylation (K_hib_) marks and negatively regulate disease resistance	Ding et al. 2012 [66], Li et al. 2021 [65], Chen et al. 2022 [56], and Chen et al. 2021 [144]
*OsBRHIS1*	Os08g0180300	Mono-ubiquitinated histone-binding protein	*M. oryzae*/Rice blasts	Suppress the expression of defense-related genes (*OsPBZc* and *OsSIRK1*) and blast resistance	Li et al. 2015 [73] and Ding et al. 2012 [66]

**Fig. 2:**
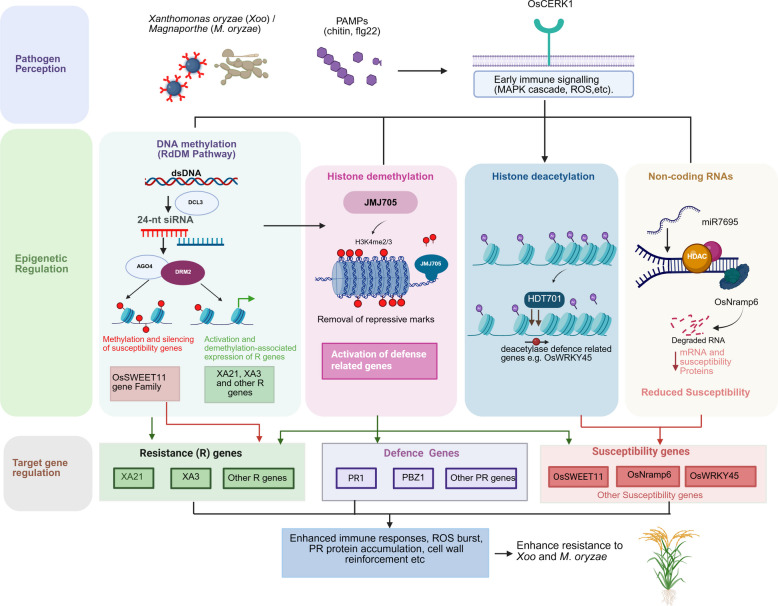
Proposed epigenetic mechanism regulating the immune response against *Xanthomonas oryzae* pv. *oryzae* (*Xoo*) and *Magnaporthe oryzae* (*M*. oryzae) in rice. (i) Recognition of the pathogen by the OsCERK1 receptor activates immune signaling. (ii) Upon detection, the RNA-directed DNA methylation (RdDM) pathway processes dsRNA into small 24-nt small interfering RNAs (siRNAs), which direct DRM2 to demethylate and activate resistance (R) genes, such as XA21, while methylating and silencing susceptibility genes, such as OsSWEET11. (iii) Histone demethylase JMJ705 prevents autoimmunity by removing histone H3 lysine 4 di- and tri-methylation (H3K4me2/3) marks from defense-related genes (e.g., PR1, PBZ1). (iv) HDT701-mediated deacetylation condenses chromatin and inhibits susceptibility factors such as OsWRKY45. (v) Non-coding RNAs (lncRNA) and microRNA (miR-7695) either recruit histone modifiers or degrade susceptibility transcripts, such as OsNramp6. Together, these epigenetic changes enhance pathogen resistance by regulating R genes (XA21, XA3) and the production of pathogen-related (PR) proteins, which activate the immune system (Created in https://www.biorender.com)

The rice blast resistance gene Pib encodes a nucleotide-binding leucine-rich repeat (NLR) protein. Its expression, low under normal conditions, is strongly induced by *M. oryzae*. Analysis revealed CG-methylation at two sites in the Pib promoter, but pathogen induction did not cause promoter demethylation [51]. Correspondingly, at the promoter region of the *PigmS* gene, DNA demethylation of MIT1 and MITE2, two tandem miniature transposons, regulates tissue-specific transcription and helps balance disease resistance and yield (Table 1) [52]. The wild type was highly susceptible to *Xoo*, likely due to Xa21G suppression. Targeting promoter regions of defense genes can increase their expression and enhance immunity [53]. Moreover, in rice, WRKY45-mediated quantitative resistance to *Xoo* is regulated by RdDM-associated pathways (Fig. 2). Consistent with its role as a key immune regulator introduced in Sect. "Epigenetic regulation of pathogen resistance", WRKY45 is further modulated by the transposon-derived siRNA TE-siR815, where Demethylation influence its expression and consequently affects susceptibility to *Xoo* [54]. In addition, epigenetic inhibitors such as 5-azacytidine can induce genome-wide DNA demethylation and activate defense-related genes, further supporting the role of DNA methylation in regulating immune responses [55].

### Histone modifications and rice immune responses

Histone modifications regulate rice immune responses. Chromatin histone changes regulate plant disease defenses [56–58]. The rice histone acetyltransferase OsHAC704 acetylates histone H3, activating defense-related genes [59]. Research has focused on Jumonji C (JmjC) domain proteins, conserved histone lysine demethylases in eukaryotes that remove di- and tri-methylations via ferrous ion- and α-ketoglutaric acid-dependent oxidative reactions [60]. Rice has 20 JmjC members (JMJ701 to JMJ720) [61], which are involved in development and biotic stress responses [62].

The jmj704 mutant, with elevated H3K4me2/3 levels, showed increased susceptibility to *Xoo* compared to the wild type, indicating a positive role in bacterial blight resistance. JMJ704 represses negative defense regulators such as OsWRKY62, Os11N3/OsSWEET14, and negative regulator of resistance (NRR) by reducing H3K27me2/3 marks. JMJ705, a histone lysine demethylase reversing H3K27me2/3, is induced by stress and pathogen infection (Fig. 2). JMJ705 overexpression conferred a leaf lesion-mimic phenotype and increased *Xoo* resistance, while loss-of-function decreased resistance. Protein gel blot analysis indicated that JMJ705 removed H3K27me3 from stress-responsive and jasmonic acid (JA)-induced genes. H3K27me3 demethylation by JMJ705 likely enhances defense gene expression, leading to necrosis and resistance [63]. *Xoo* infection induced the transcription of 15 JmjCs [62], suggesting their involvement in defense.

Rice possesses seventeen histone deacetylase (HDAC) genes [64]. Knocking down OsHDAC1 enhances broad-spectrum blast resistance without affecting plant stature, while overexpression increases susceptibility to *M. oryzae*. OsGRAS30, a novel blast resistance transcription factor, interacts with OsHDAC1, inhibiting its activity and functioning genetically upstream. This inhibition elevates H3K27ac levels, enhancing resistance. These findings integrate RNA sequencing with genome-wide identification of H3K27ac and OsHDAC1 targets (Fig. 2). Similarly, Ding et al. 2012 and Li et al. 2021 [65, 66] reported that OsHDT701 negatively regulates the defense response to *M. oryzae* and *Xoo* by interacting with the RNase P subunit Rpp30, mediating histone deacetylation at PRR and defense-related genes (Table 1).

The salicylic acid (SA)-dependent pathway primarily signals resistance to biotrophs, while the JA or ET pathways defend against necrotrophs [67]. OsSRT1, one of two homologs of rice Silent Information Regulator 2 (Sir2), epigenetically suppresses JA biosynthesis genes and Pectin Methylesterase1 (OsPME1) by deacetylation of H3K9 at their promoter [68]. Down-regulating OsSRT1 via RNA interference caused a lesion-mimic phenotype, H3K9 acetylation (H3K9ac), and hypersensitive response gene expression [69], indicating that OsSRT1 regulates pathogen resistance by modulating SA and JA pathways through histone modification.

Heterologous expression of the secreted Uv1809 protein increased rice susceptibility to bacterial blight and false smut caused by *Ustilaginoidea virens (Uv)*. Host-induced silencing of Uv1809 enhanced resistance, demonstrating its role in suppressing immunity and promoting infection. Uv1809 targets and enhances rice HDT OsSRT2-mediated histone deacetylation, reducing H4K5ac and H4K8ac levels and disrupting transcriptional activation of defense genes [70].

Small molecules inhibiting or activating histone-modifying enzymes represent another approach [71]. Trichostatin A (TSA), an HDT inhibitor, increases acetylation and gene expression. TSA treatment potentially enhances defense gene expression and disease resistance in rice [72].

Beyond canonical histone modifications, histone variants provide an additional level of epigenetic regulation by altering nucleosome composition and influencing gene expression during pathogen response. A mono-ubiquitinated protein that binds histones regulates the defense response in rice [73]. For instance, the SWI/SNF2 ATPase BRHIS1-complex in rice suppresses the expression of defense-related genes (OsPBZc and OsSIRK1) and blast resistance by interacting with mono-ubiquitinated histone variants (H2B.7 and H2A.Xa/H2A.Xb/H2A.3) in the absence of pathogen infection, indicating that the histone-binding protein helps to regulate disease resistance. Identifying additional mono-ubiquitinated histone-binding proteins in rice will develop our understanding of how histone mono-ubiquitination regulates crop-pathogen interactions.

### Non-coding RNAs and rice immunity

Non-coding RNAs, including miRNAs and long non-coding RNAs (lncRNAs), play important roles in regulating gene expression and plant immunity [74]. In rice, miRNAs regulate immune responses by targeting defense-related genes. Pathogens uniquely alter miRNAs and target expression profiles [75]. For example, rice stripe virus (RSV) infection induced more differentially expressed genes than rice dwarf virus (RDV) infection [76, 77]. Known rice miRNAs differentially expressed under normal and *M. oryzae*-infected conditions include regulators of positive resistance (miR160a, miR164a, and miR168a), negative regulators (miR396, miR827, and miR1871), and basal response regulators (miR169a, miR172a, and miR398b) [45, 78–82].

Ectopic expression clarifies miRNA function. miR7695 targets *OsNRAMP6*, which is involved in blast resistance (Fig. 2) [83]. Manipulating miRNA expression can potentially enhance defense gene expression and resistance [84]. Overexpressing Osa-miR7695 increased resistance to *M. oryzae* [44]. Similarly, miR160a targets ARF16 (ARF16), an auxin signaling component. Overexpression of miR160a enhanced resistance to *M. oryzae*, characterized by reduced fungal growth, increased H_2_O_2_ accumulation, and up-regulated defense genes [80].

miR444 targets three MIKC(C)-type MADS box proteins that suppress OsRDR1 expression by binding CArG motifs in its promoter. miR319 is essential for biotic stress responses. *M. oryzae* infection specifically induces miR319, suppressing its target *OsTCP21*. Overexpressing miR319b (miR319b-OE) inhibited key JA synthesis enzymes (lipoxygenases 2 and 5), compromising defenses [81].

miRNAs are key in virus-rice interactions. The AGO mechanism functions in small RNA activity. AGO18 overexpression confers broad-spectrum virus resistance, while its repression increases RSV susceptibility. miRNAs also upregulate RDR1, producing virus-activated siRNAs (vasiRNAs) that silence host genes to promote antiviral activity [41]. RSV infection induces miR444, which protects against infection [85]. Viruses’ infection suppresses miR528 expression, increasing the activity of its target L-ascorbate oxidase (AO), a crucial defense component generating reactive oxygen species (ROS) [86]. SPL9 activates miR528 transcription by binding motifs in its promoter, regulating the miR528-AO defense system [87].

Analysis of small RNA data across 70 plant species suggests that miRNAs regulate the evolution of *NLR* genes encoding R proteins. At least eight miRNA families target NLRs. Nucleotide diversity in target sites indicates epigenetic regulation balancing defense gene benefits and costs [88]. Artificial miRNAs (amiRNAs) offer another manipulation strategy [89]. Engineered amiRNAs can silence disease susceptibility genes [90], enhancing defense gene expression and resistance in rice. Advanced techniques such as CRISPR/Cas9 can precisely knock out specific miRNAs or lncRNAs [91].

RNA modifications represent an additional layer of epigenetic regulation that modulates gene expression at post-transcriptional levels. In *M. oryzae*, methyltransferase MTA1 (METTL4 ortholog) is essential for m6A alteration and vital for fungal virulence [92]. The loss of MTA1 impairs key processes, including appressorial penetration, autophagy and growth invasion. This disruption is linked to extensive alterations in the m6A methylation pattern and expression of autophagy-related genes, notably MoAtG8. Studies highlight the importance of MTA1-mediated m6A for regulating autophagy and pathogenicity. Furthermore, RNA modification studies have also revealed that the plant immune response during *M. oryzae* infection focuses on N4-acetylcytidine (ac4C) RNA modification [93]. Among various RNA modifications, ac4C undergoes dynamic changes and is preferentially enriched at the third codon position, where it enhances translation efficiency by stabilizing codon-anticodon interactions. Infection triggers the ac4C writer OsNAT10/OsACYR, resulting in translational reprogramming and activation of immune pathways, including JA biosynthesis. This study identifies ac4C-mediated translational regulation as a key mechanism in plant immunity. Although direct studies remain limited, RNA modifications are likely to play an important role in modulating host–pathogen interactions and represent a promising area for future research.

### Epigenetic memory and transgenerational inheritance

Epigenetic inheritance of disease resistance involves the transmission of regulatory information, such as DNA methylation, histone modifications, and small RNAs, across generations without changing the DNA sequence. Environmental conditions and genetic background influence epigenetic mark inheritance; however, evidence shows that certain epigenetic modifications can be stably inherited across generations [94]. For example, DNA methylation patterns associated with disease resistance can persist for several generations even in the absence of the initial pathogen stimulus, suggesting a potential long-term resistance mechanism.

Small RNAs have also been implicated in the transgenerational regulation of stress responses and contribute to inherited disease resistance traits [95]. Collectively, these epigenetic mechanisms provide a form of molecular memory that can maintain enhanced defense capacity in subsequent generations. Rapid and reversible epigenetic changes further allow plants to adapt to changing pathogen pressures, contributing to long-term adaptive potential in plant–pathogen interactions [96–98] and helping elucidate the evolution of defense mechanisms and future adaptive potential.

In addition to transgenerational inheritance, epigenetic mechanisms also contribute to stress memory and priming within a single generation. In plant protection, priming induces a heightened defensive state and systemic acquired resistance (SAR), enabling the rice plant to respond more effectively to future pathogen attack (Fig. 1) [99]. For example, MeJA-induced defense priming in rice enhances the H3K4me4 and H3K9ac levels at the promoter of the *OsBBPl* defense-related gene, alters genome-wide DNA methylation (5-mC) levels, and results in the creation of a chromatin-based memory of mechanical wounds [100].

Pathogen-induced epigenetic modifications in rice can also be transmitted across generations [101, 102], contributing to transgenerational disease resistance. Studies have shown that pathogen-induced epigenetic modifications in rice can be inherited, conferring enhanced disease resistance. For example, rice plants exposed to *M. oryzae* exhibit heritable DNA methylation and histone modification changes [103]. These changes activate defense genes and enhance disease resistance in progeny [104]. These findings suggest that epigenetic memory can contribute to durable disease resistance across generations. Understanding epigenetic inheritance has important practical implications for crop improvement. Identification of resistance-associated epigenetic marks enables strategies to enhance beneficial epigenetic status [105, 106]. In addition, genome and epigenome editing approaches, such as CRISPR/Cas9, may be used to establish favorable epigenetic marks [107] for developing disease-resistant rice varieties, reducing chemical pesticide dependence, and promoting sustainable agriculture [108].

## Challenges, application and future directions

### Challenges in epigenetic engineering

Despite its promise, epigenetic engineering for enhanced rice immunity faces challenges [109]. The complexity of epigenetic regulation, involving multiple interacting layers, necessitates understanding key regulatory nodes for effective manipulation [110].

Potential off-target effects from CRISPR/dCas9 systems or small molecules present another challenge [111], potentially causing unintended gene expression changes affecting growth [112]. Developing more precise tools and minimizing off-targets is crucial [113].

Epigenetic engineering offers a promising approach to enhance rice immunity by manipulating DNA methylation, histone modifications, and non-coding RNAs to improve disease resistance [114]. While challenges remain, ongoing research holds significant potential for improving crop yield and food security.

### Epigenetic biomarkers for rice disease prediction

Rice faces numerous threats, which reduce yield and quality [115]. Traditional breeding and genetic engineering have developed disease-resistant rice varieties; these methods can be time-consuming and ineffective against rapidly evolving pathogens [116]. Scientists use several techniques to improve disease resistance by underlying variation in germplasm [117], such as bisulfite sequencing (BS-Seq), whole genome bisulfite sequencing (WGBS) and methylation-sensitive amplified polymorphism (MSAP) to detect DMRs associated with disease resistance [118, 119]. Epigenetics offers a promising avenue for understanding and improving plant disease resistance [120]. Epigenetic modifications provide insights into plant responses to environmental stresses such as pathogen attacks [121]. This section explores the potential of epigenetic biomarkers for diagnosing rice diseases and predicting susceptibility or disease resistance.

Epigenetic biomarkers offer advantages over traditional methods. Epi-breeding refers to genetic breeding for epigenetic alterations, providing new opportunities for crop disease management [122]. Epigenetic studies have identified inheritable natural epialleles in rice associated with plant development and stress adaptation, highlighting rice as a key model in epigenetic breeding and genetic variations in disease resistance improvement [123]. Chemical treatment, epigenome editing, mutation in epigenetic machinery, and induced gene-specific variations could introduce epigenetic variants; for instance, as discussed earlier in DNA-methylation and disease resistance, 5-azacytidine, as an epigenetic inhibitor, can activate defense genes by demethylating genomic regions [124].

Epigenome editing and induced-gene-specific DNA methylation, histone modifications, and non-coding RNAs regulate gene expression [125], are influenced by the environment and heritability, and are valuable modifications for rice improvement. Studies on rice have identified epigenetic changes associated with disease resistance, suggesting their use as diagnostic biomarkers, such as an RNAi-inference approach, which highlights the potential of epigenetic and gene editing-based breeding, enhancing BLS by endogenously silencing the *OsROS1a* gene in rice [46, 126]. Specific DNA methylation patterns correlate with resistance to diseases such as rice blast (*M. oryzae*) and bacterial blight (*Xoo*) [127]. Resistant rice varieties often exhibit hypermethylation, which suppresses genes that facilitate pathogen invasion. Jiang et al. 2020 [128] reported map-based cloning of QTLs in which the *NBS8R* gene confers disease resistance to *Xoo*. NBS8R encodes the NB-ARC protein, which is involved in pathogen-associated molecular pattern (PAMP)-triggered immunity [129] and is regulated by non-transcription activator-like (TAL) effector XopQ-inducible Osa-mir1876 through DNA methylation as a suitable approach for epi-breeding. In rice, non-coding RNAs (miRNAs, lncRNAs) regulate gene expression and plant immunity [130]. miRNAs modulate targets by binding complementary sequences. In rice, miR393 targets auxin signaling genes exploited by pathogens; downregulating them enhances bacterial blight resistance [131]. Similarly, lncRNAs interact with chromatin modifiers, influencing the epigenetic landscape and gene expression during pathogen attacks [132].

In epigenetics, identifying molecular epigenome biomarkers involves detecting changes, validating associations with disease resistance or susceptibility, and developing diagnostic tools. Furthermore, high-throughput sequencing (bisulfite sequencing for DNA methylation, ChIP-seq for histone modifications) comprehensively profiles epiallele changes [133], identifying these DMRs and histone modification patterns by characterizing them in the early epigenetic stage in meristem/seed screening and marker-assisted selection in rice epi-breeding as a stable and heritable mark over generation [134] linked to disease resistance could be a suitable approach. Functional validation requires experimental manipulation. CRISPR-based epigenome editing alters specific DNA methylation or histone markers, assessing disease susceptibility changes [135]. Transgenic approaches overexpress or knock down non-coding RNAs to evaluate immune effects [136]. Developing diagnostic tools integrates biomarkers into assays. Quantitative PCR (qPCR) and BS-Seq detect DNA methylation changes, and ChIP-qPCR quantifies histone modifications at target genes. These assays enable rapid and accurate diagnosis of susceptibility or disease resistance in rice plants.

Apart from molecular epigenomic techniques, epigenetic modeling represents a complementary tool for rice disease resistance to fungal and bacterial pathogens by predicting the functional consequence of epigenetic variation and filling the gaps between epi-breeding and epigenetic variations [137]. Recent developments in statistical models associated with transcriptomic and DNA methylation patterns often lack mechanistic insights. In contrast, process-based models integrate biological parameters to predict how epigenetic variations influence immune-related traits. [138, 139]. In rice, such models should be used to enhance pathogen-based-disease-resistance by suppressing spontaneous epigenetic modifications and further investigate the effect of epigenetic variations by improving rice disease resistance to bacterial and fungal pathogens in epi-breeding.

Epigenetic biomarkers will provide early indicators for timely intervention. Second, epigenetic modifications are often based on their reversibility, and environmental responsiveness allows dynamic resistance regulation [140] and should be further controlled. Heritability contributes to the development of sustainable, disease-resistant rice varieties [141]. Challenges include epigenome complexity and dynamism complicating biomarker identification and validation for rice pathogens [142, 143]. Understanding the interplay between epigenetic mechanisms and their combined effects is essential. Developing cost-effective, high-throughput epigenetic diagnostic tools is critical for agricultural applications. Epigenomic biomarkers hold great promise for improving rice disease diagnosis and management. Identifying and validating resistance-associated changes enables diagnostic tools for early detection and targeted interventions. Integrating biomarkers into rice breeding programs will accelerate disease-resistant variety development, enhancing global food security. Advancing understanding of the rice epigenome will increase the potential for harnessing epigenetics to improve plant health and productivity.

## Conclusion

The current review highlights the complex epigenetic regulatory mechanisms underlying disease-resistance in rice against bacterial and fungal pathogens and discusses epigenetic biomarker approaches for developing disease-resistant rice varieties. Epigenetic processes such as DNA methylation and histone modifications are fundamental mechanisms in modulating the expression of defense-related genes. miRNAs play important roles in mediating biotic stress responses in rice, although many of their functions remain unknown. Non-coding RNAs such as miRNAs, lncRNAs, and siRNAs are key regulators in rice immunity; however, a gap persists to further explore how epigenetically regulated genes activate defense mechanisms and establish transgenerational memory for rice disease-resistance, particularly towards pathogens such as *Xoo* and *M. oryzae*. Furthermore, advances in the rice epigenome are expected to deepen our understanding of defense strategies and guide the development of disease-resistant rice varieties against bacterial pathogens.

## Data Availability

There is no data associated with the review.

## Abbreviations

*Xoo*: *Xanthomonas oryzae* pv. *oryzae*
*Xoc*: *Xanthomonas oryzae* pv. *oryzicola*
*M. oryzae*: *Magnaporthe oryzae*
*Uv*: *Ustilaginoidea virens*
miRNAs: microRNAs
siRNAs: Small interfering RNAs
dsRNA: Double-stranded RNA
DRM: Domains rearranged methyltransferases
DCL3: RNA-DEPENDENT RNA POLYMERASE: RDR DICER-LIKE 3
RdDM: RNA-directed DNA methylation
AGO: ARGONAUTE
BLS: Bacterial leaf streak
MITEs: Miniature inverted-repeat TEs
NRPD1: Nuclear RNA polymerase D1
SA: Salicylic acid
TE: Transposable element
PR: Pathogenesis-related
HDTs: Histone deacetylase tuins
HAC: Histone acetyltransferase
HDAC: Histone deacetylase
DMR: Differentially methylated region
lncRNAs: Long non-coding RNAs
Jmj: Jumonji
NRR: Negative regulator of resistance
PME1: Pectin methylesterase1
JA: Jasmonic acid
ET: Ethylene
SA: Salicylic acid
MeJA: Methyl jasmonate
BS-Seq: Bisulfite sequencing
H3K4me2/3: Histone H3 lysine 4 di- and tri-methylation
